# Identifying and regulating emotions after acquired brain injury: the role of interoceptive sensibility

**DOI:** 10.3389/fpsyg.2023.1268926

**Published:** 2023-12-21

**Authors:** Lorena Desdentado, Marta Miragall, Roberto Llorens, María Dolores Navarro, Rosa M. Baños

**Affiliations:** ^1^Polibienestar Research Institute, University of Valencia, Valencia, Spain; ^2^CIBER of Physiopathology of Obesity and Nutrition (CIBEROBN), Instituto de Salud Carlos III, Madrid, Spain; ^3^Department of Personality, Evaluation, and Psychological Treatments, University of Valencia, Valencia, Spain; ^4^Neurorehabilitation and Brain Research Group, Institute for Human-Centered Technology Research, Universitat Politècnica de València, Valencia, Spain; ^5^IRENEA, Instituto de Rehabilitación Neurológica, Fundación Vithas, Valencia, Spain

**Keywords:** interoception, interoceptive sensibility, emotional awareness, emotion regulation, acquired brain injury, depressive symptoms, depressive symptoms, alexithymia

## Abstract

**Introduction:**

Interoceptive deficits are associated with difficulties in identifying and regulating emotions. However, research on interoception after acquired brain injury (ABI) is scarce, and its relationship with emotional difficulties in this population is unknown. This study aimed to (1) examine differences in self-reported alexithymia, performance-based emotional awareness, emotion regulation, depression, and interoceptive sensibility between ABI and control individuals; and (2) analyze the role of adaptive interoceptive dimensions in these emotional processes after ABI.

**Methods:**

Forty-three individuals with ABI and 42 matched control individuals completed the Multidimensional Assessment of Interoceptive Awareness-2, the Toronto Alexithymia Scale, the Levels of Emotional Awareness Scale, the Difficulties in Emotion Regulation Scale, and the Hospital Anxiety and Depression Scale.

**Results:**

Compared to the control group, individuals with ABI showed reduced tendency to ignore unpleasant sensations increased severity of depressive symptoms, as well as tendencies to have greater difficulties in emotion regulation and lower emotional awareness. Additionally, interoceptive dimensions such as trusting, as well as not-distracting from and not-worrying about bodily sensations, played a relevant role in explaining lower alexithymia and difficulties in emotion regulation. Moreover, lower alexithymia and emotion dysregulation were related to less depressive symptoms. These relationships were invariant across ABI and control individuals.

**Discussion:**

Although individuals with ABI may have different levels of emotional abilities compared to non-ABI individuals, the relationship patterns between interoceptive and emotional processes appear to be similar between the two groups. This study suggests the potential benefit of addressing both interoceptive and emotional difficulties in treatments targeting such prevalent sequelae of ABI as depressive symptoms.

## 1 Introduction

Acquired brain injury (ABI) is an umbrella term used to refer to any damage to the brain that suddenly occurs after birth and negatively affects neurological functioning (Elbaum and Benson, 2007), with stroke and traumatic brain injury (TBI) being the most prevalent causes (FEDACE, 2015). Moreover, stroke and TBI are two of the most common causes of disability in the adult population (Feigin et al., 2014; GBD 2016 Traumatic Brain Injury and Spinal Cord Injury Collaborators, 2018). Neurocognitive disturbances after ABI have been well characterized (Barker-Collo et al., 2013; Saa et al., 2021). However, relatively little is known about emotional sequelae (Fynn et al., 2021), which impact not only the well-being of individuals with ABI, but also that of their caregivers (Freytes et al., 2021). The current study focuses on three of the most widely studied emotional processes: alexithymia, emotional awareness, and emotion regulation.

Alexithymia refers to a reduced ability to identify and express emotions, as well as engagement in externally oriented thinking (Taylor et al., 1985). Neural bases of alexithymia involve the anterior and posterior insula and the anterior cingulate cortex, among others (Kano and Fukudo, 2013; Goerlich-Dobre et al., 2014). The most widely used tool to assess this construct is the Toronto Alexithymia Scale-20 (TAS-20) (Bagby et al., 1994), which assesses subjective beliefs about one’s emotional deficits. An alternative measure is the Levels of Emotional Awareness Scale (LEAS) (Barchard et al., 2011), which is a performance-based measure that assesses the degree of specificity of emotional awareness, as indicated by the description of the emotions elicited by emotion-provoking vignettes. Therefore, the TAS-20 and LEAS represent different aspects of emotional functioning (Maroti et al., 2018). This issue is especially relevant in ABI, given that poor insight into one’s current deficits (so-called “anosognosia”) is also a common sequela of brain lesions (Prigatano, 2010). Henceforth, the term alexithymia is used to refer to self-reported difficulties in acknowledging one’s emotions (i.e., measured with the TAS-20), whereas the term emotional awareness is used to refer to the performance-based ability to be identify and label emotional states (i.e., measured with the LEAS). A recent meta-analysis showed that individuals with ABI presented higher alexithymia than neurotypical individuals, with moderate to large effects (Fynn et al., 2021). In addition, it is worth highlighting the lesion study conducted by Hogeveen et al. (2016), who observed that damage to the anterior insula was a predictor of alexithymia measured with the TAS-20 in individuals with TBI. However, to our knowledge, no previous studies have examined difficulties in emotional awareness using the LEAS in individuals with ABI compared to a control group.

Emotion regulation refers to the activation of a goal to modulate the trajectory of an emotion (Gross et al., 2011). Few studies have explored changes in the ability to regulate emotions following ABI. For instance, Cooper et al. (2015) found that individuals with ABI (vs. a control group) reported greater difficulties in acknowledging emotions, accessing regulatory strategies, and controlling their behavior when experiencing overwhelming difficulties. Similarly, Wood and Doughty (2013) found that individuals with TBI had more maladaptive coping compared to a control group.

All these disturbances in emotional processes (i.e., alexithymia, emotional awareness, and emotion dysregulation) have been proposed as transdiagnostic factors underlying psychopathology, such as depression symptoms, in several populations (Li et al., 2015; Joormann and Stanton, 2016), including ABI (Anson and Ponsford, 2006; Henry et al., 2006; Shields et al., 2016). Because suffering a stroke or TBI involves an increased risk of developing depression (Hackett and Pickles, 2014; Scholten et al., 2016), it is crucial to disentangle the mechanisms underlying these emotional deficits, which is one of the objectives of this study.

Interoception has been highlighted as an important construct involved in alexithymia, emotional awareness, and emotion regulation (Füstös et al., 2013; Smith et al., 2017; Murphy et al., 2018; Trevisan et al., 2019; Zamariola et al., 2019). Interoception is defined as the perception and integration of internal bodily signals -a process in which the insula plays a crucial role- (Khalsa et al., 2018), that includes both performance-based and self-report facets (Garfinkel et al., 2015; Khalsa et al., 2018). More specifically, interoceptive sensibility -the interoceptive feature highlighted in this study- refers to the perception of one’s bodily states in daily life, including beliefs, attitudes, and feelings about interoceptive signals (Khalsa et al., 2018). The Multidimensional Assessment of Interoceptive Awareness (MAIA) is one of the most comprehensive measures to assess interoceptive sensibility (Mehling et al., 2012, 2018). The MAIA is a self-report questionnaire that comprises eight different aspects of interoceptive sensibility: *noticing*, *not-distracting*, *not-worrying*, *attention regulation*, *emotional awareness*^1^, *self-regulation*, *body listening*, and *body trusting*.

Regarding the types of interoceptive sensibility, Mehling (2016) postulated the existence of two different attention styles towards interoceptive signals: the maladaptive and the adaptive interoceptive sensibility. Maladaptive interoceptive sensibility is characterized by catastrophization and hypervigilance towards body sensations and is related to emotional disorders. In contrast, adaptive interoceptive sensibility is characterized by attention regulation, acceptance, and respecting body sensations as emphasized in mindfulness-based approaches, and is considered a healthy, resilience-enhancing pattern. This framework highlights the relevance of interoceptive sensibility dimensions involving a non-judgmental, accepting attitude and appraisal towards bodily sensations over their subjective noticing in purely quantitative terms. Consistent with this theoretical proposal, a recent study conducted on healthy participants showed that MAIA dimensions that emphasize the adaptive appraisal of interoceptive cues play a relevant role in explaining depressive symptomatology through the mediating role of alexithymia and difficulties in emotion regulation (Desdentado et al., 2023). Specifically, it was found that *not-distracting* from and *not-worrying* about uncomfortable bodily sensations, as well as *trusting* one’s bodily cues and being able to voluntarily focus on them (*attention regulation*) were related to lower alexithymia, which in turn led to lower depressive symptomatology. Moreover, *not-worrying* was also related to better emotion regulation, which in turn led to lower depression. However, this model was tested in healthy individuals, which limits its generalization to individuals with ABI, who might exhibit high levels of depression. Moreover, emotional awareness was not studied.

In addition, only a few studies have explored interoception after ABI, and most of them investigated the link between the injured brain areas and the affected interoceptive features. For example, Grossi et al. (2014) observed that individuals with unilateral stroke showed lower interoceptive sensibility than control individuals, measured with an *ad-hoc* questionnaire, with no differences between the right and left stroke groups. Furthermore, they did not find significant associations between interoceptive sensibility and performance on a facial emotion recognition task or depressive symptoms. In contrast, Raimo et al. (2019) found that individuals with right-hemisphere stroke (vs. individuals with left brain damage and control individuals) reported lower levels of noticing of visceral interoceptive sensations. Despite this preliminary evidence, to the best of our knowledge, the effects of an ABI on interoceptive sensibility (as operationalized comprehensively with the MAIA) remain unexplored. Moreover, the link between interoceptive sensibility and the ability to identify and regulate emotions in this neurological condition is unknown. Understanding these mechanisms could suggest new therapeutic targets in the treatment of emotional difficulties in ABI.

Hence, the first aim of the present study was to examine differences in alexithymia, emotional awareness, difficulties in emotion regulation, and interoceptive sensibility between individuals with ABI and neurotypical individuals. In this regard, we hypothesized that individuals with ABI (vs. control group) would show higher levels of alexithymia, emotion dysregulation, and depression, but lower levels of emotional awareness and interoceptive sensibility (hypothesis 1). The second objective was to test whether alexithymia, emotional awareness, and difficulties in emotion regulation mediated the relationship between some adaptive interoceptive sensibility dimensions (i.e., *not-distracting, not-worrying, attention regulation, and trusting* dimensions) and depressive symptoms in individuals with ABI with path analysis, based on previous research (Desdentado et al., 2023). In this regard, it was expected that *not-distracting*, *not-worrying*, *attention regulation*, and *trusting* would predict lower alexithymia, higher emotional awareness, and lower emotion dysregulation (i.e., lack of emotional control), which, in turn, would lead to lower depression in ABI (hypothesis 2). Finally, the third objective was to test whether these associations presented similar patterns across ABI and healthy individuals. In this regard, we expected that there would not be significant differences between samples (hypothesis 3).

## 2 Materials and methods

### 2.1 Participants

A sample of individuals with stroke or TBI was recruited from the Neurorehabilitation Service of Vithas Hospital Virgen del Consuelo (Valencia, Spain) and Vithas Hospital Aguas Vivas (Carcaixent, Spain). All the potential candidates were undergoing a long-term interdisciplinary neurological rehabilitation program^2^ according to their particular needs provided by neuropsychologists, speech therapists, physiotherapists, occupational therapists, neurologists, and physical medicine and rehabilitation physicians. The inclusion criteria for this group were: age between 18 and 65 years old and moderate to good neurocognitive and communicative functioning, in order to ensure appropriate interaction and instruction-following, reflected in scores above 23 on the Mini-Mental State Examination (Folstein et al., 1975) and scores above 45 on the Mississippi Aphasia Screening Test (Romero et al., 2012), respectively. Healthy individuals were recruited as controls from the community through announcements at the university and hospital, as well as on social media. The eligibility criteria for the control group were: age between 18 and 65 years old and not having any known cognitive, psychiatric, or neurological impairments (self-reported by the participants). Individuals with a history of/current substance abuse were excluded from both groups.

A total of 207 individuals with ABI were enrolled in the rehabilitation program at the time of the study, and they were initially screened by their clinical team. Fifty-two subjects met the inclusion criteria, and 44 of them agreed to participate in the study. However, one was discharged from the Neurorehabilitation Service before being scheduled and did not participate. In the control group, 66 healthy adults also participated in the study, but 22 were eliminated from the data analyses to form a matched-control group according to basic sociodemographic characteristics, namely, sex, age, and years of education. In addition, two participants in the neurotypical group with extreme outlier scores on measures of emotional awareness and depression were removed from the analyses.

A total sample of 85 participants (43 with ABI and 42 without ABI) were included in the study. Table 1 shows the demographic characteristics of the total sample and the clinical features of the individuals with ABI.

**Table 1:** Characteristics of individuals with ABI (*n* = 43) and healthy subjects (*n* = 42).

	Individuals with ABI	Healthy individuals	*t* test		
			*t*	*p*	*Cohen’s d*
Sex (*n*, %)					
Women	14 (32.56%)	18 (42.86%)	–	–	–
Men	29 (67.44%)	24 (57.14%)			
Age (years) (*M, SD*)	44.84 (13.32)	43.74 (13.33)	0.396	0.693	0.086
Years of education, (*M, SD*)	13.72 (3.99)	14.10 (4.78)	−0.392	0.696	−0.085
Education (*n,* %)			–	–	–
Primary studies	5 (11.63%)	10 (23.81%)			
Secondary studies	12 (27.91%)	10 (23.81%)			
University studies (degree)	10 (23.26%)	12 (28.57%)			
University studies (masters)	1 (2.33%)	4 (9.52%)			
University studies (PhD)	2 (4.65%)	2 (4.76%)			
Vocational training	13 (30.23%)	4 (9.52%)			
Occupation (*n,* %)			–	–	–
Student	4 (9.76%)	4 (10.53%)			
Employed	2 (4.88%)	30 (78.95%)			
Unemployed	4 (9.76%)	1 (2.63%)			
Retired	3 (7.32%)	2 (5.26%)			
Incapacity for work	14 (34.15%)	0 (0%)			
Time off work	14 (34.15%)	1 (2.63%)			
Marital status (*n,* %)			–	.	–
Single	13 (30.23%)	8 (19.05%)			
Married/domestic partner	24 (55.81%)	30 (71.43%)			
Divorced/Separated	5 (11.63)	0 (0%)			
Widowed	0 (0%)	1 (2.38%)			
Other	2 (2.33%)	3 (7.14%)			
Etiology of the injury (*n*, %)		–	–	–	–
Traumatic brain injury	23 (53.50%)				
Ischemic stroke	9 (20.93%)				
Hemorrhagic stroke	11 (25.58%)				
Initial Glasgow Coma Scale scores in TBI individuals	5.54 (2.07)	–	–	–	–
Hemisphere damaged in stroke individuals		–	–	–	–
Left	5 (22.73%)				
Right	11 (50%)				
Bilateral	6 (27.27%)				
Time since injury (days) (*M, SD*)	27.44 (27.12)	–	–	–	–

### 2.2 Measures

#### 2.2.1 Alexithymia

Alexithymia was measured using the Toronto Alexithymia Scale-20 (TAS-20) (Bagby et al., 1994). The TAS-20 is a 20-item self-report questionnaire that comprises three dimensions of alexithymia: *difficulties identifying feelings*, *difficulties describing feelings*, and *externally oriented thinking*. In addition, an overall score ranging from 20 to 100 can be computed. Higher scores indicate more severe alexithymia. In this study, internal consistency was adequate for all scores except for *externally oriented thinking*, which was questionable (see Table 2).

**Table 2:** Differences in TAS-20, DERS, HADS, and MAIA-2 scores between individuals with ABI (*n* = 43) and healthy subjects (*n* = 42).

	*α*	*ω*	Individuals with ABI	Healthy individuals	*t* test				*Cohen’s d*		
					Statistic	*p*	95% CI		Statistic	95% CI	
							Lower	Upper		Lower	Upper
TAS-20											
Difficulties Identifying Feelings	0.86	0.87	17.80 (7.00)	16.00 (5.62)	1.32	0.768	−0.93	4.56	0.286	−0.13	0.74
Difficulties Describing Feelings	0.81	0.81	13.20 (5.13)	13.30 (4.91)	−0.04	0.961	−2.22	2.11	−0.011	−0.43	0.42
Externally Oriented Thinking	0.65	0.64	18.40 (5.19)	19.20 (4.16)	−0.78	0.878	−2.83	1.24	−0.169	−0.61	0.26
Total score	0.85	0.86	49.40 (12.9)	48.5 (11.60)	0.36	0.957	−4.34	6.27	0.079	−0.35	0.48
LEAS-A											
Self score	0.67	0.67	23.90 (4.89)	26.00 (5.38)	−1.88	0.064	−4.31	0.12	−0.408	−0.93	−0.01
Other score	0.48	0.49	22.90 (4.19)	24.80 (4.35)	−2.08	0.061	−3.77	−0.08	−0.451	−0.92	−0.04
Total score	0.71	0.71	27.90 (4.64)	30.00 (4.74)	−2.08	0.061	−4.14	−0.09	−0.451	−0.58	0.33
DERS											
Lack of emotional awareness	0.75	0.77	9.05 (3.46)	9.38 (3.29)	−0.46	0.649	−1.79	1.12	−0.099	−0.11	0.77
Lack of emotional clarity	0.78	0.79	7.98 (3.72)	7.02 (2.63)	1.37	0.220	−0.44	2.34	0.296	0.06	1.03
Nonacceptance of emotional responses	0.90	0.90	16.20 (7.94)	13.08 (6.72)	1.50	0.220	−0.78	5.58	0.326	0.13	0.96
Difficulties engaging in goal-directed behavior	0.71	0.72	11.90 (5.40)	9.50 (4.18)	2.32	0.057	0.35	4.51	0.504	0.14	1.08
Lack of emotional control	0.71	0.71	19.80 (10.30)	15.30 (5.97)	2.50	0.057	0.92	8.18	0.541	−0.33	0.51
HADS											
Depression	0.83	0.83	4.72 (4.02)	2.68 (2.78)	2.66	0.019	0.52	3.57	0.591	0.26	1.10
Rate of depression^a^: Absence of depression Possible depression	–	–	31 (77.50%) 9 (22.50%)	38 (92.68%) 3 (7.32%)	–	–	–	–	–	–	–
Anxiety	0.88	0.89	6.65 (5.45)	6.15 (4.10)	0.47	0.640	−1.64	2.64	0.105	−0.33	0.53
Rate of anxiety^a^: Absence of anxiety Possible anxiety	–	–	23 (57.50%) 17 (42.50%)	28 (68.29%) 13 (31.71%)	–	–	–	–	–	–	–
MAIA-2											
Noticing	0.67	0.68	2.59 (1.36)	2.80 (1.26)	−0.72	0.827	−0.77	0.36	−0.156	−0.57	0.28
Not-Distracting	0.69	0.74	2.85 (1.10)	2.18 (0.91)	3.06	0.024	0.23	1.11	0.665	−0.70	0.10
Not-Worrying	0.73	0.73	2.89 (1.27)	2.77 (0.95)	0.50	0.827	−0.36	0.61	0.108	−0.51	0.36
Attention Regulation	0.84	0.84	2.85 (1.30)	2.65 (1.02)	0.78	0.827	−0.31	0.70	0.169	−0.45	0.41
Emotional Awareness	0.81	0.81	3.64 (1.23)	3.79 (0.93)	−0.63	0.827	−0.62	0.32	−0.136	−0.13	0.74
Self-Regulation	0.54	0.55	2.70 (1.35)	3.16 (1.50)	−1.50	0.556	−1.08	0.15	−0.324	−0.43	0.42
Body Listening	0.68	0.70	2.34 (1.35)	2.43 (1.10)	−0.33	0.850	−0.62	0.44	−0.071	−0.61	0.26
Trusting	0.85	0.84	3.45 (1.50)	3.47 (1.17)	−0.06	0.949	−0.60	0.56	−0.014	−0.35	0.48

*α* = Cronbach’s alpha. *ω* = McDonald’s omega coefficient. ABI = Acquired brain injury. TAS-20 = Toronto Alexithymia Scale – 20 items; LEAS-A = Levels of Emotional Awareness Scale – A. DERS = Difficulties in Emotion Regulation Scale. HADS = Hospital Anxiety and Depression Scale; MAIA-2 = Multidimensional Assessment of Interoceptive Awareness – 2.

a Rates of depression and anxiety were established according to the cut-off equal or above to 8, which indicates possible cases of both depression and anxiety (Bjelland et al., 2002).

#### 2.2.2 Emotional awareness

It was assessed using the short version A of the Levels of Emotional Awareness Scale (LEAS-A) (Barchard et al., 2011). The LEAS-A consists of 10 different scenarios that involve the self and another person and are designed to elicit one basic emotion. Participants are asked to describe their own and another person’s feelings in each scenario. Scoring is based on the level of emotional awareness denoted by words and phrases attributed to the self and to the other person in the participant’s response, regardless of their appropriateness in the particular situation. A full description of the scoring procedure can be found in Barchard et al. (2011). *Self* and *other* scores range from 0 to 40, whereas the *total* score ranges from 0 to 50. In this sample, the internal consistency was adequate for the total score but questionable for the self and other scores (Table 2), similar to previous research suggesting that the total score is a better indicator of emotional awareness than individual subscales (Roberton et al., 2013).

#### 2.2.3 Emotion dysregulation

Emotion regulation was measured with the Spanish version of the Difficulties in Emotion Regulation Scale (DERS) (Gratz and Roemer, 2004; Hervás and Jódar, 2008). The Spanish version of DERS consists of 28 items that measure the extent to which they have difficulties in optimal emotion regulation. Although the original version is structured in six factors, the Spanish adaptation presented a five-factor structure, including: (1) *lack of emotional awareness* (i.e., not attending to emotions); (2) *nonacceptance of emotional responses* (i.e., the tendency to present negative secondary emotional responses to one’s own negative emotions); (3) *lack of emotional clarity* (i.e., confusion about the experienced emotions); (4) *difficulties engaging in goal-directed behavior* (i.e., interference in accomplishing tasks when experiencing negative emotions); and (5) *lack of emotional control* (i.e., difficulties in controlling one’s behavior when experiencing negative emotions and the belief that little can be done to regulate emotions effectively when feeling upset). In the Spanish translation, this dimension merged items from two original factors (“impulse control difficulties” and “limited access to emotion regulation strategies”). In this sample, internal consistency was adequate for all dimensions (Table 2).

#### 2.2.4 Depressive symptomatology

Depression was measured with the depression subscale of the Spanish version of the Hospital Anxiety and Depression Scale (HADS) (Zigmond and Snaith, 1983; Terol-Cantero et al., 2015). The HADS is a 14-item questionnaire that provides one subscore for each type of symptomatology (i.e., depression and anxiety). Higher scores indicate more severe symptomatology. In this study, the HADS showed adequate internal consistency for depression and anxiety (Table 2).

#### 2.2.5 Interoceptive sensibility

Interoceptive sensibility was measured with the Spanish version of the MAIA-2 (Mehling et al., 2018; Desdentado et al., 2023). The MAIA-2 assesses the following eight interoceptive dimensions: (1) *noticing* (i.e., tendency to be aware of one’s body sensations, regardless of their (dis)comfort); (2) *not-distracting* (i.e., tendency to not ignore uncomfortable sensations in the body or pain), (3) *not-worrying* (i.e., tendency to not worry about uncomfortable sensations in the body or pain); (4) *attention regulation* (i.e., ability to pay attention to sensations from the body); (5) *emotional awareness* (i.e., extent to which emotions are perceived as connected to bodily sensations); (6) *self-regulation* (i.e., ability to use attention to sensations from the body to regulate distress); (7) *body listening* (i.e., listening actively to the body for insight); and (8) *trusting* (i.e., degree to which the body is experienced as safe). In this sample, the internal consistency was appropriate for most of the MAIA-2 subscales but questionable for noticing, similar to previous studies (Mehling et al., 2018), and for self-regulation (Table 2).

### 2.3 Procedure

All participants were individually tested in a quiet, distraction-free room. The same experimenter tested all the participants. Once participants had been introduced to the experiment, they provided written informed consent before participating in the study. Participants self-reported their demographic information and relevant clinical data in the case of individuals with ABI, supplemented with clinical records. Afterwards, they completed several measures in the following order: MAIA-2, TAS-20, DERS, HADS, and LEAS-A. The order was configured in a way that minimized participant fatigue according to the cognitive load (from highest to lowest) estimated to be involved in completing each of the measures. Participants were seated at a desk with the questionnaires in front of them. Each statement was read aloud by the examiner, and then participants were asked to rate their response according to the Likert scale corresponding to each questionnaire, as in previous research with ABI samples (Zupan et al., 2018). In addition, to reduce the duration of the testing session and limit fatigue, oral administration of the LEAS was carried out, given that it is statistically equivalent to written administration (Roberton et al., 2013).

The study was conducted following the principles stated in the Declaration of Helsinki. The Ethics Committee at the University of Valencia approved the study (register number: 1533447).

### 2.4 Statistical analyses

Statistical analyses were performed with R 4.2.2 (R Core Team, 2022). First, descriptive analyses were conducted on sociodemographic and clinical characteristics for both ABI and neurotypical individuals, including differences between groups using two-sample t*-*tests for continuous variables and chi-square tests of independence for categorical variables.

Second, two-tailed, independent-sample t*-*tests using the *rstatix* package (Kassambara, 2021) were computed to examine differences in the study variables (MAIA-2 dimensions, TAS-20 total score, LEAS-A total score, lack of emotional control from DERS, and depression subscale from HADS) between groups. When the assumption of variance homogeneity was not met according to Levene’s test, the Welch t-test was computed. Otherwise, the Student t-test was performed. In addition, the effect size was calculated with Cohen’s *d*, with *d = 0*.20 being a small effect, *d = 0*.50 a medium effect, and *d* = 0.80 a large effect (Cohen, 1988). To control for the probability of making Type I errors due to multiple comparisons, *p*-values were adjusted using the false discovery rate procedure (Benjamini and Hochberg, 1995) with the *stats* package (R Core Team, 2022).

Third, multigroup path analyses were conducted using the *lavaan* package (Rosseel, 2012) to examine the pattern of associations specified by the theoretical model hypothesized. As a preliminary step, bivariate correlations between study variables for both ABI and healthy individuals were computed using Pearson coefficients. In addition, the models were initially computed separately for ABI and control groups, following the usual procedure carried out in previous studies using this statistical method (e.g., Miconi et al., 2017). The model included a sequence in which *not-distracting, not-worrying, attention regulation,* and *trusting* dimensions of the MAIA-2 predicted alexithymia (TAS-20 total score), emotional awareness (LEAS-A total score), and emotion dysregulation (*Lack of Emotional Control* subscale of the DERS^3^) entered as correlated, which in turn led to depressive symptomatology (HADS). All the variables were entered as manifest variables.

Then, three nested models were computed to test whether structural parameters across ABI and control individuals could be assumed as equivalent. Specifically, group invariance was tested by comparing the following hierarchical levels: (1) a baseline model in which the same structure was specified for both groups but no constraints were imposed, that is, all parameters estimates were free to vary between groups (configural invariance), (2) a model where regression paths were constrained to be the same across groups, and (3) a model where not only regression paths but also covariances were constrained to be the same across groups. Indirect effects were estimated, and the confidence intervals (CI) around the estimated effects were computed using a bootstrap resampling method, as it produces more accurate CIs than other methods (MacKinnon et al., 2007). The model was estimated using robust maximum likelihood estimation. The following criteria were used to assess the goodness of fit (Hu and Bentler, 1999): (1) the χ^2^ statistic, (2) the Comparative Fit Index (CFI), with values close to 0.90 indicating good fit (≥ 0.95 indicating very good fit), and (3) the Standardized Root Mean Square Residual (SRMR), with values close to 0.08 indicating adequate fit. To assess the goodness of fit of nested models for invariance, the χ^2^ difference test and ΔCFI equal to or smaller than −0.01 (Cheung and Rensvold, 2009) were used to not reject the hypothesis of no difference in fit between pairs of competing models, so the more constrained model can be assumed. Again, to control for the probability of making Type I errors due to the inclusion of multiple predictors, *p*-values were adjusted using the false discovery rate method, as recommended by Cribbie (2007) for structural equation models.

## 3 Results

### 3.1 Differences between ABI and control individuals in alexithymia, emotional awareness, difficulties in emotion regulation, depression, and interoceptive sensibility dimensions

Results for variance homogeneity showed that several dimensions of the DERS (i.e., *lack of emotional clarity, difficulties engaging in goal-directed behavior, and lack of emotional control*) and anxiety subscale of HADS did not meet this assumption (*p* < 0.05). Table 2 shows Welch’s t-test for these variables and Student’s t-test for the rest of the variables.

In the case of alexithymia, there were no significant differences in the TAS-20 scores between the ABI and control groups. However, individuals with ABI showed a tendency of lower emotional awareness on the LEAS-A compared to individuals without ABI. Specifically, differences in *self, other,* and *total* scores were marginally significant, all with a small (almost moderate) effect size. However, it should be noted that the *other* subscale showed low internal consistency in this study, which limits the scope of this finding.

Regarding difficulties in emotion regulation according to the DERS scores, individuals with ABI reported marginally higher scores on the *difficulties engaging in goal-directed behavior* and *lack of emotional control* subscales, both of which presented a moderate size effect. No other comparisons on the DERS subscales were statistically significant.

In addition, the ABI group presented significantly more severe depressive symptomatology (HADS Depression subscale) compared to the control group, with a moderate size effect. A chi-square test showed marginally significant differences in the rates of depression between groups (*χ^2^* = 3.70, *p* = 0.054) (Bjelland et al., 2002). However, there were no significant differences in anxiety symptoms (HADS Anxiety subscale) between the groups. Similarly, a chi-square test showed no significant differences in the rates of anxiety between groups (*χ^2^* = 1.01, *p* = 0.315) (Bjelland et al., 2002).

Regarding differences in interoceptive sensibility (MAIA-2), results showed that individuals with ABI had significantly higher scores on *not-distracting* than neurotypical individuals, indicating that the ABI group, on average, reported ignoring uncomfortable physical sensations less than the control group. This difference showed a moderate size effect. No other significant differences in the MAIA-2 dimensions were found.

### 3.2 Alexithymia, emotional awareness, and emotion dysregulation as mediators between interoceptive sensibility and depression in individuals with and without ABI

Table 3 shows bivariate correlations among the variables considered for the multivariate mediation model.

**Table 3:** Bivariate correlations between the variables included in the path-analysis models for ABI (*n* = 43) and healthy (*n* = 42) individuals.

Variable	1	2	3	4	5	6	7	8
1. Not Distracting (MAIA-2)	-	−0.36*	0.20	0.10	−0.10	−0.11	−0.08	−0.41**
2. Not Worrying (MAIA-2)	0.16	-	−0.09	0.12	−0.07	−0.16	−0.22	−0.08
3. Attention Regulation (MAIA-2)	−0.01	0.24	-	0.40**	0.20	−0.30	0.00	−0.18
4. Trusting (MAIA-2)	−0.06	−0.03	0.39**	-	0.27	−0.14	−0.14	−0.08
5. LEAS-A Total score	0.03	−0.04	−0.01	0.03	-	−0.14	0.25	0.25
6. TAS-20 Total score	−0.14	−0.20	−0.40**	−0.61**	−0.04	-	0.24	0.46**
7. Lack of emotional control (DERS)	−0.23	−0.31*	−0.29	−0.45**	−0.05	0.67**	-	0.53**
8. Depression (HADS)	−0.05	−0.20	−0.09	−0.43**	0.06	0.44**	0.51**	-

Pearson correlation coefficients displayed below the diagonal are for individuals with ABI, whereas those displayed above the diagonal are for healthy individuals. ^*^ indicates *p* < 0.05. ^**^ indicates *p* < 0.01. MAIA-2, Multidimensional Assessment of Interoceptive Awareness – 2. TAS-20, Toronto Alexithymia Scale – 20 items; LEAS-A, Levels of Emotional Awareness Scale – A. DERS, Difficulties in Emotion Regulation Scale. HADS, Hospital Anxiety and Depression Scale.

**Figure 1:**
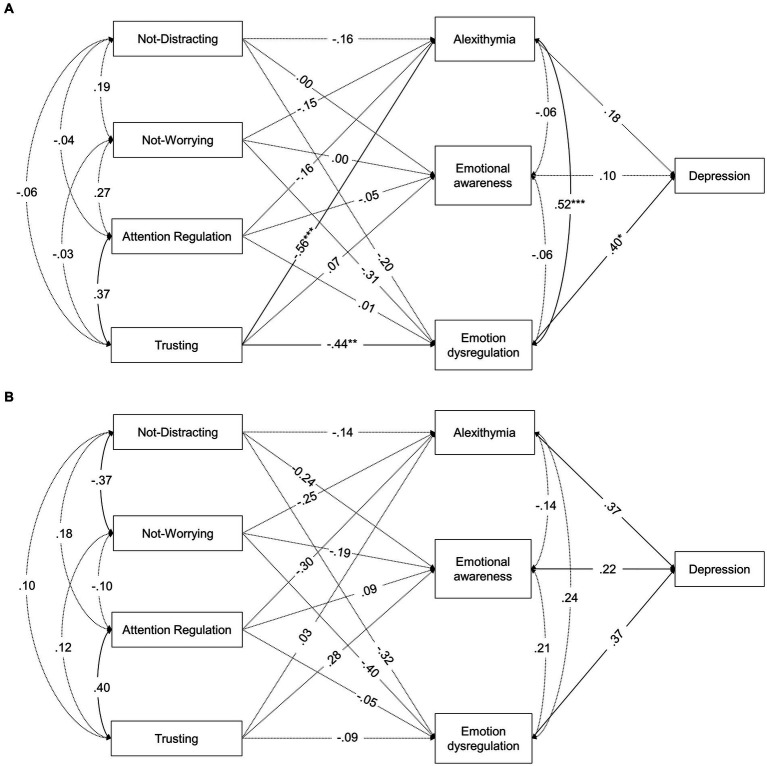
Standardized coefficients of the path-analysis model tested separately for **(A)** individuals with ABI and **(B)** healthy individuals. Continuous lines represent significant paths (*p* ≤ 0.05), whereas dotted lines represent non-significant paths (*p* > 0.05). ^*^p ≤ 0.05, ^**^ *p* ≤ 0.01, ^***^p ≤ 0.001.

Table 4 shows the fit indices for all models computed. Fit indices for the models tested separately for ABI and control groups were both adequate. Figure 1 shows their standardized path coefficients and Table 5 shows the non-standardized path coefficients.

**Table 4:** Fit indices for the sequence of models tested.

	*χ^2^* (df)	*p*	CFI	SRMR	Δ*χ^2^*	Δdf	Δ*χ^2^ -p*	ΔCFI
1. Model for individuals with ABI	4.14 (4)	0.393	0.998	0.032	–	–	–	–
2. Model for healthy individuals	6.36 (4)	0.174	0.930	0.051	–	–	–	–
3. Configural invariance model	10.50 (8)	0.232	0.975	0.037	–	–	–	–
4. Regression-constrained model	25.00 (23)	0.350	0.980	0.086	15.30	15	0.430	0.005
5. Covariance-constrained model	40.82 (32)	0.136	0.911	0.134	16.47	9	0.058	−0.069

χ^2^, Satorra−Bentler corrected Chi−square statistic. df, degrees of freedom. CFI, Comparative Fit Index. SRMR, Standardized Root−Mean − Square Residual.

**Table 5:** Non-standardized regression coefficients of separate models for ABI and control groups.

Response variable	Explanatory variable	Individuals with ABI			Healthy individuals		
		*b*	*SE*	*p*	*b*	*SE*	*p*
Alexithymia (TAS-20 Total score)	Not-Distracting	−1.897	1.435	0.401	−1.795	1.846	0.451
	Not-Worrying	−1.502	1.269	0.442	−2.996	2.383	0.314
	Attention regulation	−1.602	1.598	0.527	−3.420	1.529	0.062
	Trusting	−4.896	1.13	<0.001	0.249	1.979	0.900
Emotional awareness (LEAS-A Total score)	Not-Distracting	−0.014	0.497	0.998	−1.243	0.920	0.295
	Not-Worrying	−0.001	0.644	0.998	−0.945	0.694	0.295
	Attention regulation	−0.181	0.615	0.960	0.430	0.701	0.675
	Trusting	0.219	0.411	0.811	1.113	0.707	0.249
Lack of emotional control (DERS)	Not-Distracting	−1.829	0.885	0.117	−1.785	0.781	0.062
	Not-Worrying	−2.451	1.002	0.070	−2.113	0.920	0.062
	Attention regulation	0.096	0.975	0.998	−0.242	0.695	0.779
	Trusting	−2.933	0.919	0.007	−0.372	0.738	0.708
Depressive symptomatology (HADS)	Alexithymia (TAS-20 Total score)	0.054	0.041	0.401	0.088	0.031	0.030
	Emotional awareness (LEAS-A Total score)	0.085	0.124	0.741	0.128	0.048	0.035
	Lack of emotional control (DERS)	0.159	0.070	0.086	0.198	0.050	<0.001

*b* represents non-standardized regression coefficients. *SE*, standard error; MAIA-2, Multidimensional Assessment of Interoceptive Awareness – 2; TAS-20, Toronto Alexithymia Scale – 20 items; LEAS-A, Levels of Emotional Awareness Scale – A; DERS, Difficulties in Emotion Regulation Scale; HADS, Hospital Anxiety and Depression Scale.

Regarding associations between the IS dimensions (MAIA-2) and alexithymia (TAS-20), *trusting* was the only significant predictor in the ABI group, with higher scores of this dimension being related to lower levels of alexithymia. In addition, *attention regulation* showed a marginally significant negative relationship with alexithymia (which reached statistically significance before type I-error correction) in the control group. The 45 and 13.6% of the variance of alexithymia was explained in ABI and control individuals, respectively.

Regarding the relationships between the IS dimensions (MAIA-2) and emotional awareness (LEAS), none of them was statistically significant in either ABI or control group, the variance explained were 1 and 13.7%, respectively.

Regarding relationships between the IS dimensions (MAIA-2) and emotional dysregulation (DERS), *trusting* was the only significant predictor in the ABI group, with lower scores being related to higher lack of emotional control. None of the predictors reached statistical significance in the control group. However, *not distracting* and *not worrying* were marginally and negatively related to lack of emotional control in both groups (and they reached statistically significance before type I-error correction). The amount of its explained variance was 33.3 and 19.1% in individuals with and without, respectively.

Finally, alexithymia (TAS-20), emotional awareness (LEAS), and emotion dysregulation (LEAS) were significant predictors of higher scores on depressive symptomatology (BDI-II) in healthy individuals, whereas only emotion dysregulation was significantly related to depression in individuals with ABI. No significant indirect effects were found (Supplementary Table S1).

### 3.3 Invariance of the tested path analysis model across the ABI and the control group

**Figure 2:**
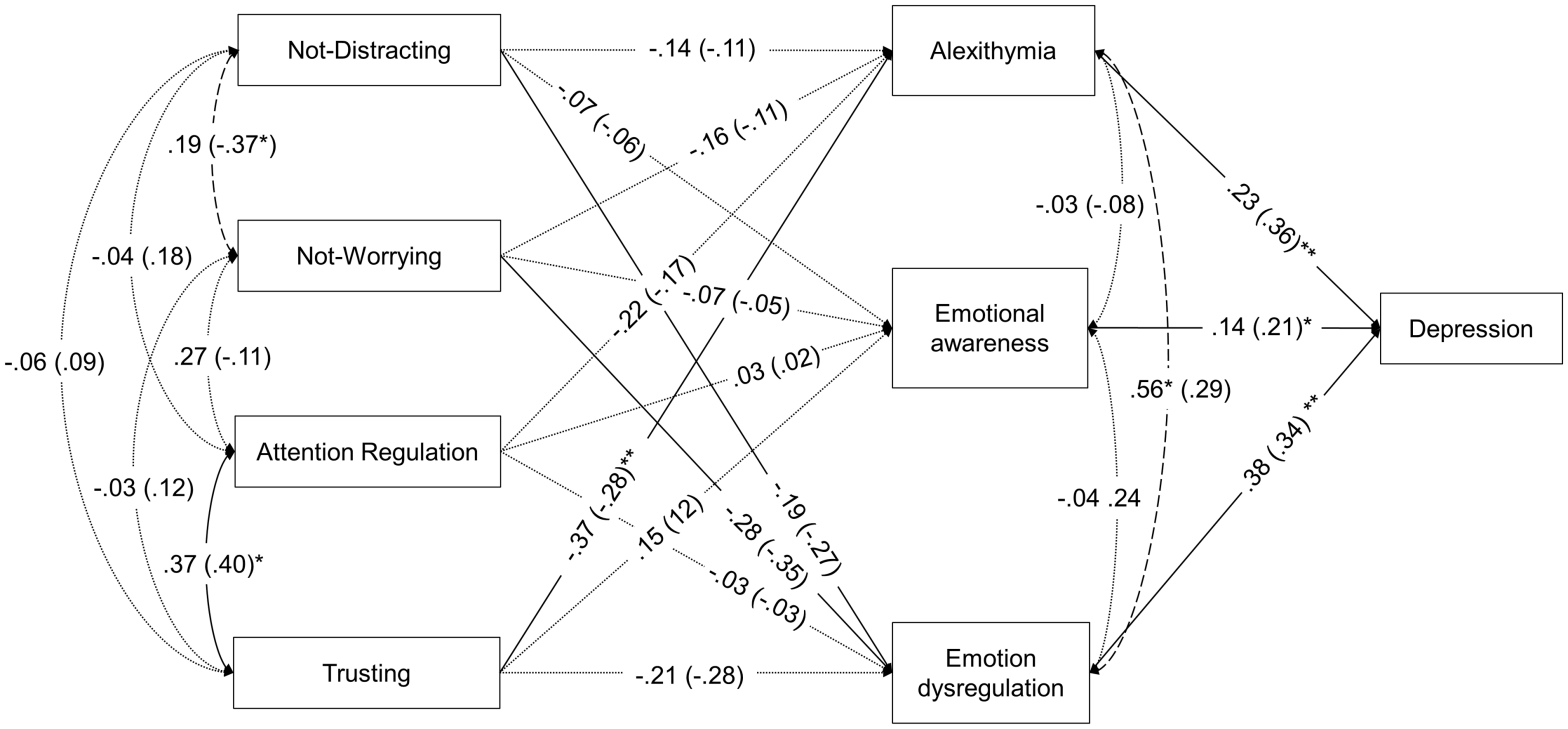
Standardized coefficients of the final multigroup path-analysis model with regression coefficients constrained. Parameters are given for individuals with ABI and then in parentheses for healthy individuals. Regression paths are indicated with single-headed arrows. Covariance paths are indicated with double-headed arrows. Continuous lines represent significant paths in both groups (*p* ≤ 0.05). Dotted lines represent non-significant paths in both groups. Dashed lines represent (covariance) paths with no-matching significance between groups (*p* > 0.05). ^*^p ≤ 0.05, ^**^p ≤ 0.01, ^***^p ≤ 0.001.

To assess whether these apparent disparities in the patterns of relationship among variables are statistically significant between the ABI and the control group, multi-group path analyses were computed. Specifically, the model invariance across ABI and control groups were calculated at three different levels: without restrictions, with constrained regression coefficients, and with constrained regression and covariances (see Statistical analyses subsection). The unconstrained model provided an acceptable fit to the data, which supports the configural invariance of the model, that is, the overall structure of relationships between the study variables is the same for both groups. The patterns of relationships found in this model were similar to those obtained in the separate models for ABI and control individuals (Supplementary Table S2). When forcing the regression coefficients to be invariant across groups, the model also showed an acceptable fit. The χ^2^ difference test between the unconstrained and constrained models was non-significant and the ΔCFI was acceptable. Therefore, the regression-constrained model can be supported, that is, the strength of the predictive relationships in the structural equation model can also be assumed to be similar for individuals with and without ABI. Finally, when further constraining covariances to be equal across groups, the fit model was not significantly worse than the one with only regression constraints according to the χ^2^ difference test, but it was worse according to the ΔCFI criterion. In addition, the SRMR was above the acceptable limit for a good fit. Therefore, it was rejected the null hypothesis that both ABI and healthy individuals showed invariant covariance relationships (i.e., correlations between the MAIA-2 dimensions and correlations between the mediators in the model) in the model. It should be noted that *not-distracting* and *not-worrying* were negatively related in the control group, but not in the ABI group (see Table 3 and both Figures 1, 2), which could explain the differences in covariances between groups. Thus, the last acceptable model, that is, the regression-constrained model, was adopted.

Figure 2 shows standardized coefficients and Table 6 shows non-standardized path coefficients for this final model. Regarding associations between the interoceptive sensibility dimensions (MAIA-2) and alexithymia (TAS-20) found in the model with invariant regression coefficients across groups, the only significant predictor was *trusting*, with higher levels of *trusting* being related to lower levels of alexithymia. Specifically, a one-point increase in *trusting* was associated with a 2.94-point decrease in TAS-20 total scores. Overall, the model explained 29.7% of the variance in alexithymia in the ABI group and 17.3% in the control group.

**Table 6:** Non-standardized regression coefficients of the model with constrained regression coefficients across ABI and control groups.

Response variable	Explanatory variable	*b*	*SE*	*p*
Alexithymia (TAS-20 Total score)	Not-Distracting	−1.50	1.17	0.303
	Not-Worrying	−1.46	1.07	0.282
	Attention regulation	−2.05	1.17	0.146
	Trusting	−2.94	1.11	0.024
Emotional awareness (LEAS-A Total score)	Not-Distracting	−0.30	0.48	0.657
	Not-Worrying	−0.26	0.49	0.687
	Attention regulation	0.10	0.47	0.841
	Trusting	0.46	0.43	0.393
Lack of emotional control (DERS)	Not-Distracting	−1.58	0.55	0.019
	Not-Worrying	−1.98	0.70	0.019
	Attention regulation	−0.21	0.57	0.768
	Trusting	−1.32	0.68	0.109
Depressive symptomatology (HADS)	Alexithymia (TAS-20 Total score)	0.08	0.03	0.019
	Emotional awareness (LEAS-A Total score)	0.12	0.05	0.027
	Lack of emotional control (DERS)	0.17	0.04	<0.000

*b* represents non-standardized regression coefficients. *SE*, standard error.

Regarding the relationships between the interoceptive sensibility dimensions (MAIA-2) and emotional awareness (LEAS) in the final model, none of them was statistically significant in either group, and the explained variance of emotional awareness was 3.8% for individuals with ABI and 1.7% for individuals without ABI.

Regarding relationships between the interoceptive sensibility dimensions (MAIA-2) and emotional dysregulation (DERS), the significant predictors were *not-distracting* and *not-worrying*, with lower scores on these dimensions (i.e., trying to ignore and worrying more about uncomfortable bodily sensations) being related to higher levels of *emotional dysregulation*. Specifically, a one-point increase in *not-distracting* was associated with a 1.58-point decrease in *emotional dysregulation,* and a one-point increase in *not-worrying* was associated with a 1.98-point decrease in *emotional dysregulation*. Overall, the amount of explained variance of lack of emotional control was 17.5% for the ABI group and 25.1% for the control group.

Finally, alexithymia (TAS-20), emotional awareness (LEAS), and emotion dysregulation (DERS) were significant predictors of higher scores on depressive symptomatology (BDI-II). Specifically, one-point increases in alexithymia, emotional awareness, and emotional dysregulation were associated with 0.08-, 0.12-, 0.17-point increases in depressive symptomatology, respectively. Overall, the amount of explained variance of depression was 31.9% for ABI individuals and 39.4% for healthy individuals. No significant indirect effects were found (Table 7).

**Table 7:** Standardized parameter estimates, standard errors, p-values, and 95% confidence intervals of the indirect effects in the final path-analysis model.

Explanatory variable (MAIA-2)	Mediating variable	*β*	*SE*	*p*	Lower 95% CI	Upper 95% CI
Not-Distracting	Alexithymia (TAS-20)	−0.03	0.03	0.294	−0.09	0.03
Not-Worrying		−0.04	0.03	0.254	−0.10	0.03
Attention regulation		−0.05	0.04	0.206	−0.13	0.03
Trusting		−0.09	0.05	0.078	−0.18	0.01
Not-Distracting	Emotional awareness (LEAS-A)	−0.01	0.02	0.603	−0.05	0.03
Not-Worrying		−0.01	0.02	0.630	−0.05	0.03
Attention regulation		0.00	0.02	0.861	−0.04	0.04
Trusting		0.02	0.02	0.334	−0.02	0.06
Not-Distracting	Emotion dysregulation (DERS)	−0.07	0.04	0.046	−0.14	0.00
Not-Worrying		−0.10	0.05	0.046	−0.21	0.00
Attention regulation		−0.01	0.03	0.742	−0.08	0.05
Trusting		−0.08	0.06	0.159	−0.19	0.03

MAIA-2, Multidimensional Assessment of Interoceptive Awareness - 2; TAS-20, Toronto Alexithymia Scale – 20 items; LEAS-A, Levels of Emotional Awareness Scale – A; DERS, Difficulties in Emotion Regulation Scale; *β*, Standardized estimate for the indirect effects. *SE*, standard error; CI, Confidence interval.

## 4 Discussion

The current study aimed (1) to explore differences in alexithymia, emotional awareness, emotion regulation, depressive symptoms, and interoceptive sensibility between ABI and control individuals and (2) to examine the role of adaptive interoceptive sensibility dimensions in these emotion-related variables after ABI.

First, our findings showed that participants with ABI tended to show lower emotional awareness and emotion regulation abilities and more severe depressive symptoms than their matched controls. These results are consistent with previous studies indicating that ABI usually involves deficits in emotional processing (Cooper et al., 2015; Maza et al., 2020; Quemada, 2020). Similarly, a large body of research highlights the high prevalence rate of psychopathological disturbances in this clinical condition, including depressive symptomatology (Hackett and Pickles, 2014; Scholten et al., 2016).

In contrast, no differences were found in self-reported alexithymia (TAS-20) between participants with and without ABI. Although some studies also showed discrepant results (Bossu et al., 2009; Wood and Doughty, 2013), a meta-analysis by Fynn et al. (2021) concluded that individuals with ABI show higher scores on the TAS-20 than individuals without ABI. The absence of significantly higher scores on the TAS-20 in our ABI sample could be due to the possible influence of other variables. In this regard, Fynn et al. (2021) highlighted two metacognitive processes that can be affected in individuals with ABI: anosognosia and anosodiaphoria. This means that some individuals with ABI might have difficulties in recognizing their deficits (i.e., anosognosia) or show indifference to the consequences of their deficits (i.e., anosodiaphoria). Given the low scores on the LEAS-A (i.e., the performance-based emotional awareness), we can speculate that the ABI group might have difficulties in recognizing their deficits (anosognosia) and, consequently, reported low alexithymia on the TAS-20. Regarding anosodiaphoria, the ABI group may have had fewer problems with this process because their DERS scores suggest that they were aware of the interference of their negative emotions in their daily lives. Hence, further research is needed to determine the interactions between metacognitive abilities, interoception, and emotional functioning after ABI and confirm these assumptions.

Regarding the interoceptive sensibility dimensions, individuals with ABI reported less distraction from uncomfortable physical sensations than neurotypical individuals. These differences in the *not-distracting* subscale may be explained by the fact that all the participants with ABI were involved in a multidisciplinary rehabilitation process in which they received constant clinical supervision. These participants are commonly asked about their physical, cognitive, and emotional condition, which might have increased their self-monitoring and made them more aware of any odd bodily sensations. No other differences in the interoceptive sensibility dimensions were found between the two groups. The absence of significant differences could again be explained by anosognosia, so that individuals with ABI could have interoceptive sensibility deficits that were unaware of and, consequently, were not reported. In particular, this possibility would only explain dimensions of the MAIA-2 that directly involve metacognitive awareness of one’s abilities, such as *noticing, emotional awareness, attention regulation,* and *self-regulation.* However, it is not plausible that anosognosia is involved in the lack of differences in the interoceptive sensibility dimensions that focus more on appraisal or attitudes towards one’s own body (e.g., *trusting*). Future studies should consider how metacognitive abilities affect interoception in people with ABI.

Our second objective was to determine whether lower levels of some interoceptive sensibility dimensions (i.e., those that largely reflect an adaptive attentional style towards interoceptive cues) are the initial step in a “cascade” that leads to depressive symptomatology through the mediating effect of alexithymia, emotional awareness, and emotional dysregulation in individuals with ABI. Contrary to our second hypothesis based on previous research on healthy individuals (Desdentado et al., 2023), our results did not support these indirect effects. However, the pattern of direct effects between the variables was partially consistent with what was expected, as explained in the following paragraphs.

Moreover, the findings of this study allow us to assume this pattern of relationships to be equivalent between individuals with and without ABI, supporting the third hypothesis. Regarding the relationships found in both groups, we found that *trusting* in one’s body emerged as a negative predictor of alexithymia; that is, those individuals who had less confidence in their body showed higher alexithymia. These associations are consistent with previous studies showing that *trusting* in bodily sensations is one of the interoceptive sensibility aspects that has been most consistently related to decreased emotional disturbances and greater well-being in different populations (Brown et al., 2017; Zamariola et al., 2018; Schmitz et al., 2021). Moreover, *not distracting* from and *not-worrying* about uncomfortable bodily signals was associated with lower emotion dysregulation, which is also congruent with the findings by Desdentado et al. (2023). Taken together, these findings are in line with the existing evidence on the positive effects of mindfulness-based approaches on emotional processing (Wu et al., 2019), given that some core components of mindfulness refer to attending to the present experience and bodily states with a non-judgmental attitude even if they are unpleasant (as opposed to experiencing it as something to worry about or be wary of).

However, *attention regulation* (i.e., the ability to sustain and control attention to body sensations), which is also a crucial aspect of mindfulness, did not reach statistical significance in explaining alexithymia, emotional awareness, or emotion dysregulation in the final model. In contrast, it was marginally significant in the initial model conducted only in the control group, similar to findings of previous work (Desdentado et al., 2023). The lack of evidence for this association in individuals with ABI could also be affected by the clinical care they received including the monitoring of physical sensations (e.g., numbness, paresthesia, pain, etc.), which might encourage them to pay attention to bodily sensations for medical purposes, but not related to emotional processing.

It also should be noted that, although the explained variance of both alexithymia and emotion dysregulation was substantial, the interoceptive sensibility dimensions included in the model did not contribute to explaining emotional awareness as assessed by the LEAS -a performance-based measure- in either individuals with or without ABI. This result may be due to the lack of correspondence between self-reported and performance-based measures, which has been previously and widely found in the general and ABI populations (e.g., Garfinkel et al., 2015; Lau et al., 2021), as also occurs with the report by significant others (Prigatano et al., 1998). More research is needed to examine the relationships between interoception and emotional awareness by using both self-report and performance-based measurements of both constructs and thus disentangle how the type of measure affect the link found between them.

Finally, alexithymia, emotional awareness, and emotion dysregulation were significantly related to depressive symptomatology in the final model, explaining a substantial amount of variance in both groups. Although the relationships between alexithymia and emotion dysregulation and depression were in the expected direction, the finding that higher levels of emotional awareness were associated with more depressive symptomatology was inconsistent with our initial hypothesis. Bailen et al. (2019) found that depressive severity was associated with an increased likelihood of having meta-emotional experiences, particularly negative (secondary) emotions about negative (primary) emotions. Since the LEAS-A scoring system does not differentiate between emotions and meta-emotions, nor does it differentiate between the appropriateness or valence of the emotions described by participants in the test, it is possible that those participants who showed greater specificity in their emotional awareness according to the LEAS (and thus, scored higher) were also those who showed more negative meta-emotional experiences, which might explain this finding. Future studies should jointly examine the role of emotional awareness and the presence of positive and negative meta-emotions in depression to better understand these relationships.

### 4.1 Clinical implications and future directions

This is the first study to reveal the potential role of interoception in regulating emotions in individuals with ABI. In light of these preliminary findings, some clinical implications can be tentatively proposed. First, our results show that neuropsychological programs designed to rehabilitate the consequences of ABI should pay special attention to the ability to regulate emotions to decrease depressive symptomatology, as therapeutic target. Second, our study provides some insights into interoceptive processes that should be taken into consideration in treatments designed to improve difficulties in emotion regulation after ABI. Specifically, the experience of the body as a safe and trustworthy place, and attending body signals without being distracted and worry, seems to be a key ingredient of good emotion regulation skills after ABI, which contributes to less depressive symptomatology. Future research would benefit from investigating whether interventions that include enhancement of these interoceptive dimensions have an effect on improving emotion regulation skills in individuals with ABI. Initial attempts have been made to improve the identification and regulation of emotions considering body-related components in individuals with ABI. For instance, Neumann et al. (2017) conducted a phase I trial to test the acceptability and initial efficacy of a treatment targeting emotional awareness and emotion regulation that included a lesson focused on interoceptive awareness, showing promising effects. However, the design of this study did not allow the authors to unravel the contribution of each treatment component or establish causal relationships regarding its effects. In addition, preliminary research suggests that mind–body interventions can be helpful and acceptable for individuals with ABI (Combs et al., 2018; Niraj et al., 2020; Acabchuk et al., 2021). However, research in this field is still in its infancy, and the efficacy and mechanisms of change of these approaches to ABI should be further explored.

### 4.2 Limitations

This study has several limitations. First, a convenience sample was recruited whose size was relatively small, which might have led to the results being underpowered. Second, the ABI group included adult individuals with brain damage due to distinct etiologies, namely, stroke and TBI. Moreover, the specific location of the brain lesion in the clinical group was not documented, nor was the current pharmacological therapy. Given that both interoceptive deficits and alexithymia have shown consistent neural correlates including the anterior insula and the anterior cingulate cortex, among others (Kano and Fukudo, 2013; Goerlich-Dobre et al., 2014; Khalsa et al., 2018), future studies should examine weather affection in these areas also influences the relationships between these constructs. Third, the ability to regulate emotions was measured exclusively in a self-reported manner. Although the ABI group reported greater difficulties in regulating their emotions than the control group, their responses could still be somewhat biased due to anosognosia and/or anosodiaphoria, which might have influenced the validity of the results reported in this study. Future studies should include performance-based tasks to assess emotion regulation skills in individuals with ABI. Fourth, the cross-sectional the design of the current study does not allow to establish causal relationships. Future studies should adopt longitudinal and experimental designs to determine the directionality of the relationships examined herein. In addition, long-term prospective studies would allow to investigate the role of aging (and its interaction with ABI) in these associations. Fifth, although our clinical sample was screened to exclude individuals with severe impairments in cognitive and communicative functioning, this was not comprehensively assessed in this study. In other words, cognitive processes such as perception, memory, or working memory that were not assessed in this study could affect the measure outcomes (Salas et al., 2019). This limits the extent to which our findings can be generalized to individuals with ABI with specific sequelae. In addition, a detailed description of the motor and sensory functioning of the participants was not included in this study. Future studies should examine how sensorimotor impairments in ABI affect interoceptive functioning. Finally, other interoceptive features (e.g., interoceptive accuracy) were not included in this study and might also play a crucial role in alexithymia, emotional awareness, emotion regulation, and depressive symptoms in ABI, as observed in previous studies (Füstös et al., 2013; Eggart et al., 2019). Future research should explore the relationships between other performance-based interoceptive aspects and emotional deficits following ABI.

### 4.3 Conclusion

To conclude, our study showed that individuals with ABI exhibit a trend of worse performance on emotional awareness, greater emotion regulation difficulties, and more severe depressive symptomatology, and they reported being less distracted from their body signals than neurotypical individuals. In contrast, there were no significant differences between groups in self-reported alexithymia. Despite these mean differences in the study variables between individuals with and without ABI, this study revealed that interoceptive sensibility, emotional skills, and depressive symptomatology showed a pattern of relationship after ABI similar to that found in healthy individuals. Specifically, experiencing the body as a safe and trustworthy place was the most relevant interoceptive sensibility dimension in explaining lower alexithymia, whereas not ignoring and not worrying about uncomfortable bodily signals were significantly associated with fewer difficulties controlling negative emotions. Furthermore, lower levels of alexithymia, greater emotion awareness, and lack of emotional control were related to higher depressive symptomatology.

## Data availability statement

The dataset and the analytic code used in this study are publicly available. This data can be found here: https://osf.io/kb6jp/.

## Ethics statement

The studies involving humans were approved by Ethics Committee at the University of Valencia. The studies were conducted in accordance with the local legislation and institutional requirements. The participants provided their written informed consent to participate in this study.

## Author contributions

LD: Conceptualization, Data curation, Formal analysis, Investigation, Methodology, Validation, Writing – original draft. MM: Conceptualization, Formal analysis, Methodology, Supervision, Validation, Writing – review & editing. RL: Project administration, Supervision, Validation, Writing – review & editing. MN: Investigation, Project administration, Validation, Writing – review & editing. RB: Conceptualization, Methodology, Project administration, Supervision, Writing – review & editing.

## Footnotes

^1^It should be noted that the MAIA dimension called “emotional awareness” does not correspond to the conceptualization of emotional awareness as measured by the LEAS or to alexithymia as measured by the TAS-20. Specifically, in the MAIA-2, “emotional awareness” assesses the perceived link between one’s own emotions and body states. Therefore, this MAIA subscale should not be confused with the ability to recognize and describe emotional experiences.

^2^Criteria for admission to the long-term outpatient adult neurorehabilitation program of Vithas Hospitals are: (1) aged above 16; (2) having a diagnosis of an ABI as evidenced by neuroimaging (i.e., computed tomography scan or magnetic resonance imaging) regardless of the etiology and severity, (3) being medically stable, and (4) being susceptible to benefit from neurorehabilitation treatment according to an initial clinical evaluation. The average length of stay for patients ranges from 3 months to several years, depending on the patient’s needs and insurance coverage.

^3^The TAS-20 and LEAS total scores are widely considered appropriate indices of alexithymia and emotional awareness, respectively (Roberton et al., 2013; Preece et al., 2018). However, the use of a DERS total score is not recommended because second-order models produce a weak fit (Fowler et al., 2014). In addition, some of its dimensions conceptually overlap alexithymia, such as the *lack of emotional awareness* and *lack of emotional clarity* subscales. Therefore, we focused on the *lack of emotional control* subscale of the DERS as an index of emotion dysregulation in the structural equation model, as in a previous study conducted by Desdentado et al. (2023).
