# Supplementary material for: Identifying and regulating emotions after acquired brain injury: the role of interoceptive sensibility
Source: Front Psychol. 2023 Dec 21;14:1268926. doi: 10.3389/fpsyg.2023.1268926 (PMC10764614; doi:10.3389/fpsyg.2023.1268926)
Supplement: Supplementary file 1 [file Data_Sheet_1.docx]

**Table S1.**

*Standardized parameter estimates, p-values, and 95% confidence intervals of the indirect effects in the path analysis models separately for ABI and healthy individuals*

| Explanatory variable (MAIA-2) | Mediating variable | ABI individuals | | | | Healthy individuals | | | |
| --- | --- | --- | --- | --- | --- | --- | --- | --- | --- |
|  |  | *β* | *p* | Lower 95% CI | Upper 95% CI | *β* | *p* | Lower 95% CI | Upper 95% CI |
| Not-Distracting | Alexithymia (TAS-20) | -.029 | .317 | -.091 | .034 | -.052 | .414 | -.176 | .072 |
| Not-Worrying |  | -.027 | .354 | -.088 | .035 | -.091 | .272 | -.254 | .071 |
| Attention regulation |  | -.028 | .398 | -.104 | .048 | -.110 | .155 | -.263 | .042 |
| Trusting |  | -.100 | .232 | -.278 | .078 | .009 | .907 | -.147 | .166 |
| Not-Distracting | Emotional awareness (LEAS-A) | .000 | .978 | -.028 | .027 | -.052 | .302 | -.152 | .047 |
| Not-Worrying |  | .000 | .998 | -.038 | .038 | -.042 | .272 | -.117 | .033 |
| Attention regulation |  | -.005 | .766 | -.043 | .033 | .020 | .602 | -.056 | .096 |
| Trusting |  | .007 | .632 | -.027 | .041 | .061 | .182 | -.028 | .150 |
| Not-Distracting | Emotion dysregulation (DERS) | -.081 | .097 | -.192 | .030 | -.116 | .125 | -.264 | .032 |
| Not-Worrying |  | -.127 | .148 | -.313 | .058 | -.145 | .088 | -.311 | .022 |
| Attention regulation |  | .005 | .922 | -.112 | .122 | -.018 | .749 | -.126 | .090 |
| Trusting |  | -.176 | .098 | -.398 | .045 | -.031 | .658 | -.170 | .107 |

*Note.* MAIA-2 = Multidimensional Assessment of Interoceptive Awareness - 2; TAS-20 = Toronto Alexithymia Scale – 20 items; LEAS-A = Levels of Emotional Awareness Scale – A; DERS = Difficulties in Emotion Regulation Scale. ABI = Acquired brain injury

**Table S2.**

*Standardized and non-standardized regression coefficients of the configural invariance model across individuals with ABI and healthy individuals*

| Response variable | Explanatory variable | *ABI group* | | | | *Control group* | | | |
| --- | --- | --- | --- | --- | --- | --- | --- | --- | --- |
|  |  | *b* | *SE* | *β* | *p* | *b* | *SE* | *β* | *p* |
| Alexithymia (TAS-20 Total score) | Not-Distracting | -1.90 | 1.44 | -.16 | .186 | -1.80 | 1.85 | -0.14 | .331 |
|  | Not-Worrying | -1.50 | 1.27 | -.15 | .236 | -3.00 | 2.38 | -0.25 | .209 |
|  | Attention regulation | -1.60 | 1.60 | -.16 | .316 | -3.42 | 1.53 | -0.30 | .025 |
|  | Trusting | -4.90 | 1.13 | -.56 | <.001 | 0.25 | 1.98 | 0.03 | .900 |
| Emotional awareness (LEAS-A Total score) | Not-Distracting | -0.14 | 0.50 | .00 | .978 | -1.24 | 0.92 | -0.24 | .177 |
|  | Not-Worrying | 0.00 | 0.64 | .00 | .998 | -0.95 | 0.69 | -0.19 | .173 |
|  | Attention regulation | -0.18 | 0.62 | -.05 | .768 | 0.43 | 0.70 | 0.09 | .540 |
|  | Trusting | 0.22 | 0.41 | .07 | .595 | 1.11 | 0.71 | 0.28 | .116 |
| Lack of emotional control (DERS) | Not-Distracting | -1.83 | 0.89 | .20 | .039 | -1.79 | 0.78 | -0.32 | .022 |
|  | Not-Worrying | -2.45 | 1.00 | -.32 | .014 | -2.11 | 0.92 | -0.40 | .022 |
|  | Attention regulation | 0.10 | 0.98 | .01 | .921 | -0.24 | 0.70 | -0.5 | .727 |
|  | Trusting | -2.93 | 0.92 | -.44 | .001 | -0.37 | 0.74 | -0.09 | .614 |
| Depressive symptomatology (HADS) | Alexithymia (TAS-20 Total score) | 0.05 | 0.04 | .18 | .187 | 0.09 | 0.03 | 0.37 | .004 |
|  | Emotional awareness (LEAS-A Total score) | 0.09 | 0.12 | .10 | .494 | 0.13 | 0.05 | 0.22 | .007 |
|  | Lack of emotional control (DERS) | 0.16 | 0.07 | .40 | .023 | 0.20 | 0.05 | 0.37 | <.001 |

*Note. b* represents non-standardized regression coefficients. *SE* = standard error. *β* represents standardized regression coefficients.
